# Waste Orange Peels as a Source of Cellulose Nanocrystals and Their Use for the Development of Nanocomposite Films

**DOI:** 10.3390/foods12050960

**Published:** 2023-02-24

**Authors:** Francesco Bigi, Enrico Maurizzi, Hossein Haghighi, Heinz Wilhelm Siesler, Fabio Licciardello, Andrea Pulvirenti

**Affiliations:** 1 Department of Life Sciences, University of Modena and Reggio Emilia, 42015 Reggio Emilia, Italy; francesco.bigi@unimore.it (F.B.); enrico.maurizzi@unimore.it (E.M.); hossein.haghighi@unimore.it (H.H.); fabio.licciardello@unimore.it (F.L.); 2 Department of Physical Chemistry, University of Duisburg-Essen, 45141 Essen, Germany; hw.siesler@uni-due.de; 3 Interdepartmental Research Centre for the Improvement of Agri-Food Biological Resources (BIOGEST-SITEIA), University of Modena and Reggio Emilia, 42015 Reggio Emilia, Italy

**Keywords:** bioactive packaging, renewable nanomaterials, agrifood waste, cellulose nanocrystals

## Abstract

To date, approximately 30–50% of food is wasted from post-harvesting to consumer usage. Typical examples of food by-products are fruit peels and pomace, seeds, and others. A large part of these matrices is still discarded in landfills, while a small portion is valorized for bioprocessing. In this context, a feasible strategy to valorize food by-products consists of their use for the production of bioactive compounds and nanofillers, which can be further used to functionalize biobased packaging materials. The focus of this research was to create an efficient methodology for the extraction of cellulose from leftover orange peel after juice processing and for its conversion into cellulose nanocrystals (CNCs) for use in bionanocomposite films for packaging materials. Orange CNCs were characterized by TEM and XRD analyses and added as reinforcing agents into chitosan/hydroxypropyl methylcellulose (CS/HPMC) films enriched with lauroyl arginate ethyl (LAE^®^). It was evaluated how CNCs and LAE^®^ affected the technical and functional characteristics of CS/HPMC films. CNCs revealed needle-like shapes with an aspect ratio of 12.5, and average length and width of 500 nm and 40 nm, respectively. Scanning electron microscopy and infrared spectroscopy confirmed the high compatibility of the CS/HPMC blend with CNCs and LAE^®^. The inclusion of CNCs increased the films’ tensile strength, light barrier, and water vapor barrier properties while reducing their water solubility. The addition of LAE^®^ improved the films’ flexibility and gave them biocidal efficacy against the main bacterial pathogens that cause foodborne illness, such as *Escherichia coli*, *Pseudomonas fluorescens*, *Listeria monocytogenes*, and *Salmonella enterica*.

## 1. Introduction

The public’s knowledge about the negative environmental impact of traditional plastics is quickly expanding [1]. In this context, biodegradable films have emerged as valuable solutions able to guarantee the safety of food and prolong food shelf life, thus reducing the environmental impact of plastic disposal [2,3].

Among biopolymers applied for the production of packaging, hydroxypropyl methylcellulose (HPMC) and chitosan (CS) have received much attention due to their biocompatibility, abundance, nontoxicity, and excellent film-forming features [4,5]. HPMC is a semi-synthetic cellulose-derived ether, mainly obtained from renewable sources such as wood pulp and cotton linters [6]. It was approved as a human food additive by the European Parliament and Council (Directive No. 95 No. 95/2/EC) and the US Food and Drug Administration (FDA, 21 CFR 172.874) [6]. Recently, this cellulose derivative has been employed for the production of packaging items since it forms flexible, odorless, transparent, and oil-resistant films. Even so, HPMC-based films show high sensitivity to moisture, limiting their massive application in the food sector [7]. CS is a cationic polymer obtained through the partial N-deacetylation of chitin, the second most abundant polysaccharide in nature after cellulose [8]. Chitin is a linear molecule formed by N-acetyl-D-glucosamine units linked via *β* (1,4) glycosidic bonds. It represents one of the main structural constituents of the exoskeletons of crustaceans and mollusks. Additionally, fungal biomass represents another major source of chitin [9]. Recently, CS has been regarded as a prospective candidate for engineering applications [10]. It is soluble in acidic aqueous solutions due to the protonation of the NH_2_ groups. It inhibits the growth of a wide range of micro-organisms due to its cationic behavior. Furthermore, CS possesses high film-forming properties, and CS films exhibit good barrier properties to gases such as CO_2_ and O_2_. However, they have high production costs and low mechanical properties [11]. Due to the mutual compatibility of these polymers [12], previous studies suggested that blending CS and HPMC could represent a feasible strategy for improving their single functional properties and reducing their drawbacks [4,5,13].

A current trend in research consists of reinforcing the biopolymer matrix with nanomaterials (i.e., bio-nanocomposites) to overcome the technical criticisms of packaging [14]. In fact, nanomaterials can contribute to organizing the polymer matrix in a dense nanoscale network through hydrogen bonding linkages, enhancing the optical, physical, barrier, and mechanical performances of the packaging system [15,16]. The derived packaging materials can be further functionalized with biodegradable compounds (either natural or synthetic) with strong antimicrobial and/or antioxidant activities. These combined techniques (i.e., nanoreinforcement and bioactive addition) make it possible to create a new generation of active packaging solutions, reducing the overall use of traditional food preservatives and producing functional packaging films with an extended range of applications in the food packaging sector [8,17,18].

Among nanomaterials, cellulose nanocrystals (CNCs) have been widely studied as reinforcing agents for different biopolymers due to their renewability, biocompatibility, and low cost [19]. CNCs are nanosized cellulosic crystals with a needle-shaped morphology, high aspect ratio (10–70), high specific surface area (~150 m^2^/g), and low density (~1.6 g/cm^3^). They are conventionally obtained by partially dissolving cellulose fibrils through sulfuric acid hydrolysis. In this method, sulfuric acid esterifies the surface hydroxyl groups of cellulose, whose amorphous regions are easily hydrolyzed and removed. Meanwhile, the crystalline regions, which are more resistant, remain intact. This biobased nanomaterial presents several prospective applications from different fields, which range from reinforcing agents for films and nanocomposites to drug delivery systems, tissue engineering materials, and medical implants [20].

In the last few years, many efforts have been dedicated to isolating valuable compounds such as CNCs from agricultural by-products and waste (i.e., sustainable cellulosic feedstocks), aiming to shift from a linear to a circular economy model in the agrofood sector [21]. In fact, the application of these by-products allows for the improvement of rural economies without jeopardizing other supplies. Specifically, CNCs were extracted from different agricultural by-products, including pineapple peels [19], grape pomace [22], tomato peels [23], sugarcane bagasse [24], banana peels [25], and orange peels [26]. The results of these works (e.g., the yield of extraction; the structure of the crystals, etc.) drastically changed in accordance with the parameters of extraction, and the plant source [27]. In this context, orange by-products discarded by the juice processing industry were highlighted as one of the most promising sources of CNCs due to their high cellulosic content, and their extended availability [26,28].

The purpose of this study was to provide an optimized protocol for the extraction of cellulose from discarded orange peels and its conversion to CNCs. CS/HPMC films were strengthened by the addition of extracted CNCs as fillers. These bio-nanocomposites were enriched with lauroyl arginate ethyl (LAE^®^), a synthetic cationic surfactant characterized by a broad biocidal activity, biodegradability, nontoxicity, and prospective applicability as a packaging additive [29,30]. In addition, considering the potential use of these films as innovative food packaging systems, the effects of CNCs (10% *w*/*w* of biopolymer) and/or LAE^®^ (5% *w*/*w* of biopolymer) on the optical, physical, microstructural, water barrier, mechanical, and antimicrobial properties of the CS/HPMC film were assessed.

## 2. Materials and Methods

### 2.1. Materials and Reagents

Macè s.r.l. provided the orange peels (Ferrara, Italy). Acros Organics^TM^ (Geel, Belgium) provided CS (molecular weight 100–300 kDa). ACEF SPA provided HPMC (hydroxypropyl 5–8%, methoxy 28–30%) (Piacenza, Italy). Glycerol (99.5%) was purchased from Merck (Darmstadt, Germany). Ethanol, acetic acid, sulfuric acid, sodium hydroxide, hydrogen peroxide, sodium dodecyl sulfate, EDTA, and cetyltrimethylammonium bromide were provided by Sigma-Aldrich (St. Louis, MO, USA). Vedeqsa (Barcelona, Spain) provided LAE^®^ (Mirenat^®^ NSF). Brain heart infusion agar (BHIA), and brain heart infusion broth (BHIB) were purchased from Biolife (Milan, Italy).

### 2.2. Pre-Treatment and Chemical Composition Analysis of Orange Peels

Fresh orange peels were dried to 10 ± 0.5% RH in an infrared oven (ZTM Mechatronic, Reggio Emilia, Italy) at 50 °C. The dry peels were then milled to obtain orange peel powder, which was vacuum-packed and stored at −18 °C. Moisture, protein, fat, and ash contents of orange peel powder were measured according to AOAC methods [31]. Van Soest’s [32] modified protocols were applied to determine cellulose, hemicellulose, lignin, and nonfiber components. The analyses were performed with a crude fiber extractor, FIWE6 (VELP Scientifica, Velate, MB, Italy). Each analysis was performed in triplicate.

### 2.3. Cellulose Isolation

Cellulose was obtained from dry orange peel following a five-step procedure, as depicted by Coelho et al. [15] with slight modifications. The sample/solvent ratio was maintained constant at 1:20 (*w*/*v*) for each step. Briefly, dry orange peel (50 g) was mixed with a 50% ethanolic solution (60 °C, 5 h, 400 rpm) to remove sugars, vitamins, pigments, and polyphenols. The solid material was recovered by centrifugation (4800× *g*, 30 min, 20 °C) (Remi Elektrotechnik Ltd., Vasai, India), dried, and treated with a 1 M HCl solution (70 °C, 5 h, 400 rpm) to separate acid-soluble polysaccharides and polyphenolic compounds. The material was rinsed with double-distilled water to neutralize the pH before being treated with a 5% NaOH solution (90 °C, 6 h, 400 rpm) to dissolve hemicelluloses and lignin, and then bleached twice with 5% H_2_O_2_ (pH 11.5, 60 °C, 6 h) to remove residual lignin and phenolic monomers. The final product was washed to neutral pH and freeze-dried (1.35 Pa, −50 °C for 24 h) through a lyophilizer (Labconco Corporation, Kansas City, MO, USA). The extraction yield (%) was obtained by dividing the extracted dry mass of cellulose by the amount of dry orange peel.

### 2.4. Production of Cellulose Nanocrystals (CNCs) and Yield Calculation

According to Coelho et al. [22], CNCs were extracted using sulfuric acid hydrolysis. Orange cellulose (3 g) was mixed with a 64 wt% sulfuric acid solution (1:30 ratio, *w*/*v*) at 45 °C for 60 min (500 rpm). Diluting the mixture with cold distilled water (1:15 ratio, *v*/*v*) stopped the hydrolysis. CNCs were precipitated by centrifugation (4800× *g*, 45 min, 20 °C) (Remi Elektrotechnik Ltd., Vasai, India), and rinsed with deionized water four times (4800× *g*, 15 min, 20 °C). The washing cycles were followed by ultrasonication (28 kHz, 180 W, 3 min) (Argo Lab, Carpi, Italy) to disrupt large CNCs aggregates. The resulting suspension was dialyzed to neutral pH against double-distilled water using a cellulose dialysis membrane with a 10–12 kDa molecular weight cutoff (Sigma-Aldrich, Milan, Italy), and then freeze-dried (−50 °C, 1.35 Pa) to produce CNCs powder. The dry mass of CNCs was then divided by the dry mass of orange cellulose to compute the production yield (%).

### 2.5. Preparation of Film-Forming Solutions and Nanocomposite Films

Two grams of CS were dissolved in 100 mL of a 1% acetic acid solution at 55 °C for 60 min to produce CS film-forming solution (FFS) [33]. HPMC FFS was produced by mixing 2 g of HPMC in 100 mL distilled water at 80 °C for 60 min [34], and cooled to room temperature. Both FFSs were enriched with glycerol (30% *w*/*w* of the polymer) as a plasticizer. Then, CS and HPMC FFSs were mixed (1:1 *w*/*w* ratio) to prepare a CS/HPMC blend [4]. LAE^®^ (5% *w*/*w* of the polymer) and/or CNCs (10% *w*/*w* of the polymer) were separately added to the CS/HPMC FFSs [15,30,35]. These concentrations were selected based on preliminary tests, which highlighted that higher concentrations of CNCs and LAE^®^ could have compromised the structural integrity of the films (e.g., formation of aggregates) without any functional benefit. Meanwhile, no relevant effects on the technical performances of the films had been observed with lower concentrations of CNCs and LAE^®^. To ensure that these chemicals were uniformly distributed throughout the polymer matrix, ultrasonication (28 kHz, 180 w, 30 min) (Argo Lab, Carpi, Italy) and further stirring (500 rpm, 30 min) were used. The obtained FFSs were degasified with a vacuum pump (Vacuumbrand GMBH + CO KG, Wertheim, Germany) at 70 kPa for 15 min. Then, 20 mL of FFS were poured onto petri dishes (14.4 cm in diameter) to create the films, which were then dried overnight (45% RH; 25 °C).

### 2.6. Transmission Electron Microscopy (TEM) and X-ray Diffraction (XRD)

TEM was used to describe the shape and size of CNCs. The experiments were carried out using a Talos F200S G2 microscope (Thermo Scientific, Brno, Czech Republic), running at an acceleration voltage of 200 kV. Briefly, 0.2 g of CNCs were mixed with 5 mL of double-distilled water with ultrasonication (28 kHz, 180 W, 20 min) (Argo Lab, Carpi, Italy). A drop of CNCs suspension was pipetted onto a formvar/carbon-coated copper grid (200 mesh). TEM micrographs were analyzed by means of ImageJ software (v. 1.53a, National Institutes of Health, Bethesda, Maryland, MD, USA). The average width and length of 100 randomly chosen CNCs were measured.

Pure orange CNCs and films were subjected to Cu-Kα radiation at 40 kV and 40 mA in an X-ray diffractometer (X’Pert Pro, PANalytical, Eindhoven, The Netherlands) to capture the XRD patterns. At room temperature, each sample was inspected with a scanning angle of 2θ from 5 to 110 at a rate of 2.45°/min. According to Nam et al. [36], Equation (1) was used to calculate the crystallinity index (CI):

Crystallinity index (%) = (I_cry_ − I_am_/I_cry_) × 100 (1)

where I_cry_ is the peak’s greatest intensity of diffraction in the crystalline region, which occurs at an angle of 2θ∼22.5° for cellulose I and ∼21.7° for cellulose II. At an angle of 2θ∼18° for cellulose I and ∼16° for cellulose II, I_am_ is the minimal intensity in the amorphous region [37,38].

### 2.7. Scanning Electron Microscopy (SEM)

A field emission scanning electron microscope (Nova NanoSEM 450, FEI, Hillsboro, OR, USA) was used to obtain the films’ surface and cross-section morphologies. Double-sided tape was used to mount the cut film samples (2 × 2 mm^2^) on the stainless steel stub. An acceleration voltage of 10 kV was used throughout the analysis, which was conducted in a low vacuum mode (80 Pa).

### 2.8. Attenuated Total Reflection (ATR)/Fourier-Transform Infrared Spectroscopy (FT-IR)

An ATR/FT-IR spectrometer was used to measure the infrared spectra (Alpha, Bruker Optik GmbH, Ettlingen, Germany). By combining 64 scans, the spectra were captured in the wavenumber range of 4000–400 cm^−1^ at a resolution of 4 cm^−1^. Three copies of each determination were created.

### 2.9. Thickness and Mechanical Properties

The thickness of each film was measured at five different spots using a digital micrometer (IP65, SAMA Tools, Viareggio, Italy). According to ASTM D882-12, a dynamometer (Z1.0, ZwickRoell, Genova, Italy) coupled with a 1 kN loading cell was used to test the films’ elongation (E%), Young’s modulus (YM), and tensile strength (TS) [39]. The crosshead speed was 10 mm/min, and the initial grip separation was 70 mm. The TestXpert^®^ II software (V3.31) (ZwickRoell, Genova, Italy) was used to record the E% (%), YM (MPa), and TS (MPa).

### 2.10. UV-Vis Light Transmittance, Opacity, and Color

The UV barrier characteristics of the films were assessed at UV wavelengths of 200, 280, and 350 nm, and visible wavelengths of 400, 500, 600, 700, and 800 nm. As described by Haghighi et al. [40], the optical parameters were calculated using film samples of 2 × 2 cm^2^ and a spectrophotometer (VWR^®^ Double Beam UV VIS 6300 PC 152 spectrophotometer, VWR International Srl, Milan, Italy). The film’s opacity was estimated using Equation (2):

Opacity value = −LogT_600_/d (2)

where d is the thickness (mm) of the film and T_600_ is the transmittance at 600 nm. Four measurements were obtained, and average values were computed.

Using a CR-400 Minolta colorimeter (Minolta Camera, Co., Ltd., Osaka, Japan) with a D65 illuminant and a 10° observer angle, the coordinates L* (lightness), a* (redness/greenness), and b* (yellowness/blueness) were determined. A white standard with the following calibration values was used: L* = 99.36, a* = 0.12, and b* = 0.06. Using Equation (3), the total color variation (∆E*) was calculated:

(3) $${\Delta E}^{*}=\sqrt{{\left({\Delta L}^{*}\right)}^{2}+{\left({\Delta a}^{*}\right)}^{2}+{\left({\Delta b}^{*}\right)}^{2}}$$

where the variances between the relevant color parameters of the samples and a white standard used as the film background are denoted by letters ∆L*, ∆a*, and ∆b*, respectively. Ten measurements were taken for each film.

### 2.11. Water Content (WC) and Water Solubility (WS)

According to Equation (4), the WC was recorded after drying the films (2 × 2 cm^2^) in an oven (ZTM Mechatronic, Reggio Emilia, Italy) at 105 ± 2 °C for 24 h:

WC (%) = ((W_0_ − W_1_)/W_0_) × 100 (4)

where W_0_ and W_1_ are film weights (g) before and after drying.

The WS was determined following the protocol described by Gontard et al. [41], with slight modifications. The samples (2 × 2 cm^2^) were dried to a constant weight in an oven (ZTM Mechatronic, Reggio Emilia, Italy) set at 105 ± 2 °C to determine the initial dry weight of each film (W_i_). After being submerged in 50 mL of distilled water for 24 h at 25 °C, each film was then dried to a constant weight in an oven (ZTM Mechatronic, Reggio Emilia, Italy) at 105 ± 2 °C (W_f_). The WS was determined using Equation (5):

WS (%) = ((W_i_ − W_f_)/W_i_) × 100 (5)

where W_i_ and W_f_ stand for the films’ initial and final dry weights (g) of the films, respectively. The tests were carried out in triplicate.

### 2.12. Water Vapor Permeability (WVP)

The film’s water vapor permeability (WVP) was assessed using a modified version of the ASTM E96 method [39]. Glass test cups with an interior diameter of 10 mm and a depth of 55 mm were sealed on top with the film samples. Two grams of anhydrous CaCl_2_ had been previously placed in the test cups (0% RH). The cups were incubated at 45 °C in desiccators with BaCl_2_ to ensure a relative humidity of 90%. For a week, the cups were weighed daily to ensure steady-state permeation. Linear regression was used to determine the slope of the mass gain with time. According to Equations (6) and (7), WVTR (g/day·m^2^) and WVP (g/kPa·day·m^2^) were calculated.

WVTR = ΔW/Δt × A (6)

WVP = WVTR × L/ΔP (7)

where ΔW/Δt stands for the weight growth as a function of time (g/day), A for the film’s surface area (m^2^), L for its mean thickness (mm), and ΔP for the vapor pressure gap across the film (kPa). WVTR analyses were carried out in triplicate.

### 2.13. In Vitro Antimicrobial Activity 

According to Haghighi et al. [30], the disk diffusion assay was used to evaluate the film’s biocidal activity against four common food-borne bacterial pathogens: *Escherichia coli* (ATCC 43888), *Pseudomonas fluorescens* (ATCC 13525), *Listeria monocytogenes* (ATCC 19115), and *Salmonella enterica* sv. Typhimurium (ATCC 14028). Each pure strain’s loop was placed into 10 mL of sterile BHIB and incubated at 30 °C for 24 h. Sterilized BHIA plates were streaked with 100 µL of inoculum that contained 10^6^ CFU/mL of each strain of bacteria. Films were cut into disks with a diameter of 18 mm, sterilized with UV-C irradiation, and then put on the inoculated plates’ surfaces. The plates were incubated at 30 °C for 24 h. The pictures were taken with a reflex Canon mod. EOS 700D (Canon, Tokyo, Japan), and ImageJ v1.53e (National Institutes of Health, Bethesda, MD, USA) was used to measure the diameters of the inhibition zones. Each analysis was performed in triplicate.

### 2.14. Statistical Analysis

A one-way analysis of variance (ANOVA) was used to assess the recorded data’s statistical significance. SPSS statistical software (SPSS 20 for Windows, SPSS Inc., IBM, New York, NY, USA) was used to evaluate the difference between the means with a post hoc Tukey’s multiple range test (*p* < 0.05). The outcomes were presented as a mean ± standard deviation of the measured values.

## 3. Results and Discussion

### 3.1. Chemical Analysis of Orange Peel, CNC Yield, and Visual Appearance

**Figure 1:**
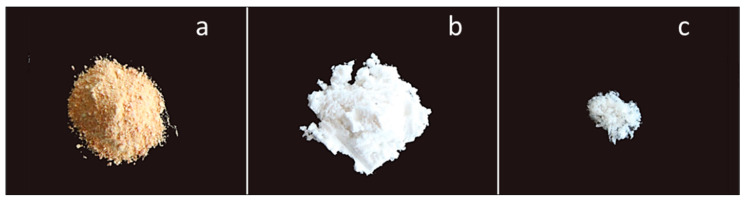
The visual appearance of (**a**) raw orange peel powder, (**b**) orange peel cellulose, and (**c**) cellulose nanocrystals.

The chemical analysis showed that dry orange peel (Figure 1a) contained 9.8 ± 0.2% moisture, 2.0 ± 0.1% crude fat, 5.9 ± 0.3% protein, and 2.75 ± 0.2% ash. A total of 64.2 ± 3.1% nonfibrous carbohydrates, 0.2 ± 0.01% hemicelluloses, 1.0 ± 0.08% lignin, and 14.2 ± 0.2% cellulose was determined. This preliminary step of analysis aimed to estimate the potential yield in CNCs from the starting matrix, and to set the purification steps aiming to remove the chemical impurities [27].

The orange peel powder was subjected to a combined treatment including ethanolic extraction, acid washing, alkali washing, and two repeated bleaching steps (H_2_O_2_) to isolate cellulose (Figure S1 in Supplementary Materials) [15]. A total of 5.8 g of material was obtained from 50 g of dry orange peel, consisting of a fine white powder (Figure 1b) mainly composed of cellulose (86.4%). The extraction yield (~11.5%) was comparable to that of the citrus (Kinnow) peel reported by Naz et al. [26]. Overall, the combined extraction route allowed for effective cellulose extract from orange peel powder. This step was reported to reduce the amorphous character of cellulose, improving its reactivity to the subsequent acid treatment [22]. Orange peel cellulose was then hydrolyzed with 64 wt% sulfuric acid for 60 min to generate CNCs with a 27% yield. These outcomes were similar to those observed by Coelho et al. [22], who reported CNCs yields of 27.56% and 20.96% for pomegranate peel cellulose treated with acid hydrolysis for 30 and 60 min, respectively. These values were higher than those described in previous research. As an example, Lu and Hsieh [42] reported a yield of 6.4% for CNCs isolated from rice straw cellulose. As well, Jiang and Hsieh [23] reported a yield of isolation of 15.7% for tomato peel cellulose, using similar conditions. Many factors may affect the CNC’s yield, such as reaction time, acid concentration, cellulose amount per acid volume, and the cellulose purity.

The aqueous CNC suspension was transparent and stable, probably due to the sulfate groups introduced by sulfuric acid hydrolysis [23]. Dry CNCs appeared as light and well-distributed crystals (Figure 1c).

### 3.2. Transmission Electron Microscopy (TEM) and X-ray Diffraction (XRD)

**Figure 2:**
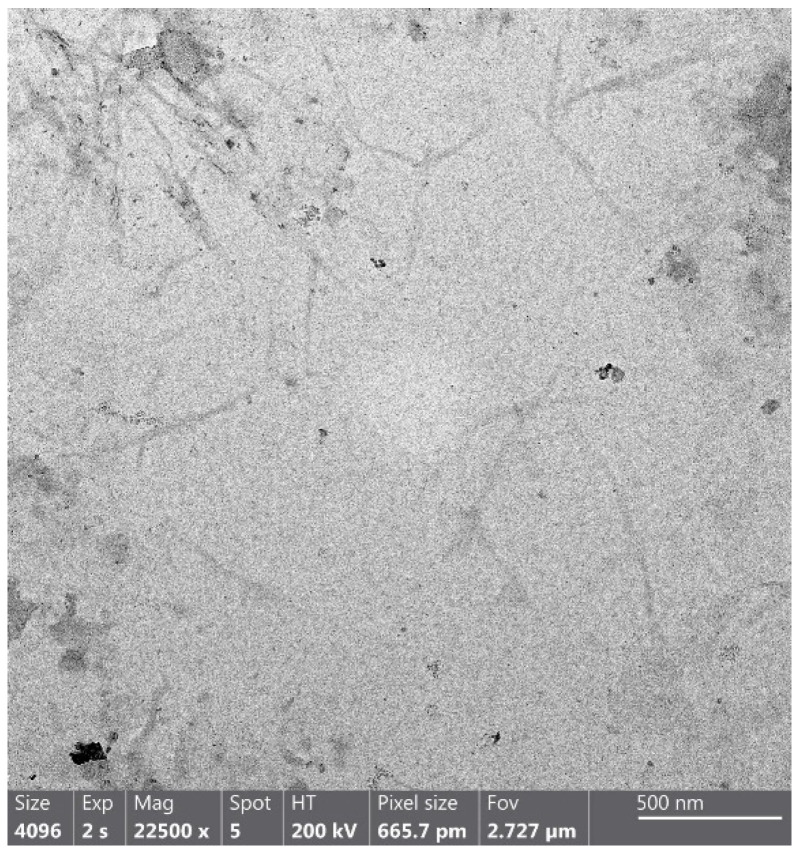
TEM image of cellulose nanocrystals (CNCs) from orange peels.

Orange CNCs’ shape and size were determined using TEM (Figure 2). CNCs displayed morphology resembling needles. The nanoparticles had an aspect ratio of 12.5, which was computed from an average of 500 nm to 40 nm in length and width. These results agreed with previous studies, highlighting that plant CNCs have a length of 100–500 and a width of 5–70 nm. Authors suggested that plant source and acid hydrolysis parameters significantly influence the characteristics of CNCs [43,44].

**Figure 3:**
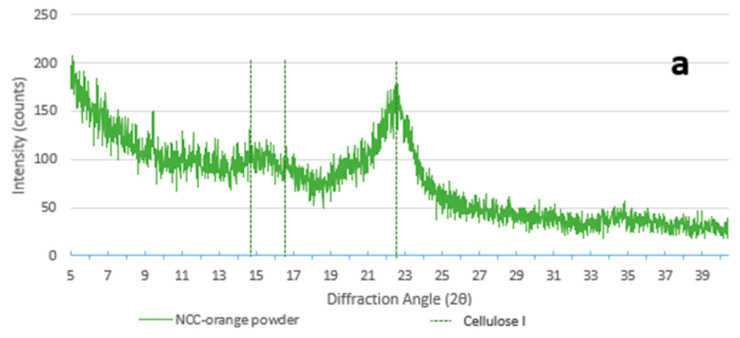

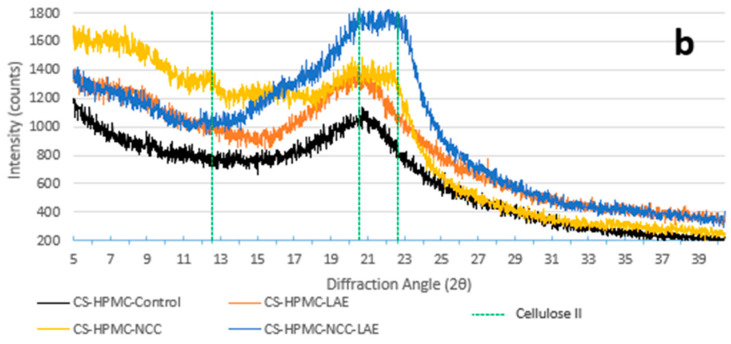
XRD patterns’ signals of (**a**) CNCs from orange peel, and (**b**) films with and without CNCs.

XRD patterns of CNCs (Figure 3a) showed cellulose I polymorphism, due to the presence of the typical diffraction peaks at the angles of 14.5° (1 $\bar{1}$ 0), 16.5° (1 1 0), and 22.5° (2 0 0) [45], with a crystallinity index (CI%) of 61.93% (Table 1). This result value was lower than in previous studies carried out with the same technique [38]. It is conceivable to imagine that cellulose isolation via alkaline treatment may have resulted in a decrease in the value of CI% by weakening the crystalline structure and increasing the amorphous region [46]. In the presence of CNCs, the formation of new bonds between cellulose and the polymers caused an evident shift towards cellulose II polymorphism, with a visible peak at 12° (1 $\bar{1}$ 0), 20° (1 1 0), and 22° (0 2 0) [45]. The CI% of the films added with CNCs (24.96–26.81%) was consistently higher than that of those without nanocrystals (7.42–7.65%), as shown in Table 1. The films (Figure 3b) that contain only the CS/HPMC blend and LAE^®^ depict a state that lies in between cellulose polymorphisms I and II.

### 3.3. Characterization of CS/HPMC Nanocomposite Films

#### 3.3.1. Surface and Cross-Section Morphology

**Figure 4:**
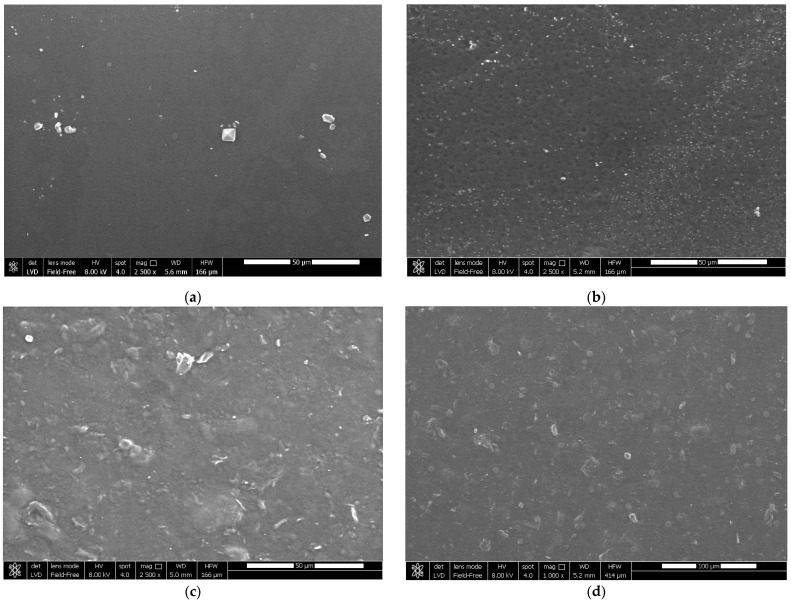
SEM images (surface) of films based on (**a**) chitosan/hydroxypropyl methylcellulose blend (CS/HPMC), (**b**) CS/HPMC/LAE^®^, (**c**) CS/HPMC/CNCs, and (**d**) CS/HPMC/CNCs/LAE^®^.

**Figure 5:**
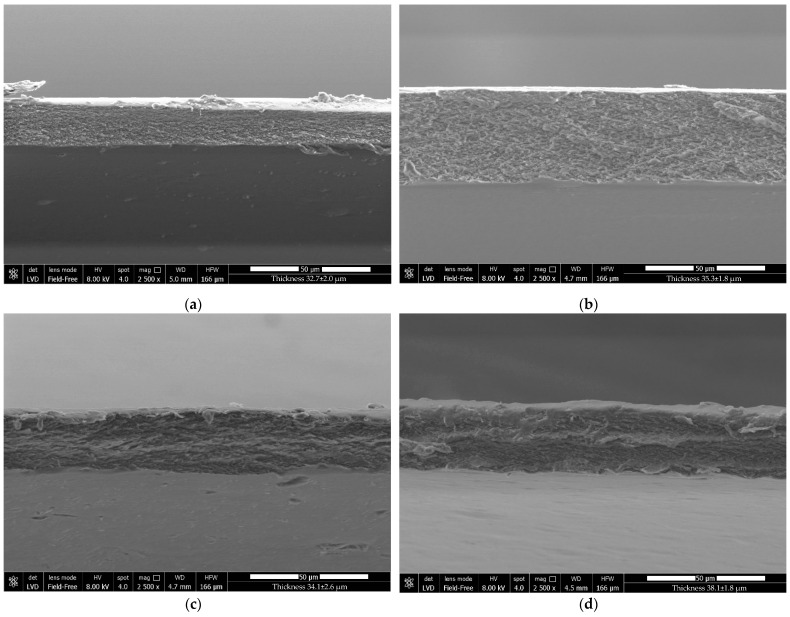
SEM images (cross-section) of films based on (**a**) chitosan/hydroxypropyl methylcellulose blend (CS/HPMC), (**b**) CS/HPMC/LAE^®^, (**c**) CS/HPMC/CNCs, and (**d**) CS/HPMC/CNCs/LAE^®^.

The optical, physical, mechanical, and barrier properties of a film are directly affected by the microstructure of the film, which is dependent on the miscibility and compatibility of the film’s elements [47]. Figure 4 and Figure 5 show the surface and cross-section of films created using a CS/HPMC blend as the control, those reinforced with CNCs, and/or those enriched with LAE^®^.

The control film’s surface was homogeneous, smooth, and compact (Figure 4a). Phase separation was not observed in the CS/HPMC blend, suggesting that associative connections made CS and HPMC extremely compatible [13]. Similar to the CS/PVA blend films enriched with LAE^®^ described by Haghighi et al. [30], active films enriched with LAE^®^ (5% *w*/*w* biopolymer) (Figure 4b) revealed a loosely compact surface with small pores and irregularities. The partial release of this component from the film’s surface may be the cause of this result. Figure 4c,d show a rough surface with small particles for CS/HPMC/CNCs and CS/HPMC/CNCs/LAE^®^, respectively. The strong linkages between the CNCs’ hydroxyl groups, which encouraged the partial aggregation of the CNCs on the film surface, were likely the cause of this phenomenon [48]. However, it was shown that even with a relatively high concentration of CNCs, they served as fillers in the films’ structures and had good dispersion in the matrix [8].

The compact microstructure of the control film can be deduced by analyzing the cross-section (Figure 5a), demonstrating the excellent affinity between CS and HPMC. The film containing LAE^®^ (Figure 5b) showed a continuous and compact microstructure, suggesting a high level of miscibility between the polymer matrix and this active compound. The structure of the CNC-reinforced films was slightly asymmetrical and sponge-like (Figure 5c,d). This result confirmed the predominance of nanocrystal-nanocrystal associations over nanocrystal-polymer interactions in the polymer matrix.

#### 3.3.2. ATR/FT-IR Spectroscopy

**Figure 6:**
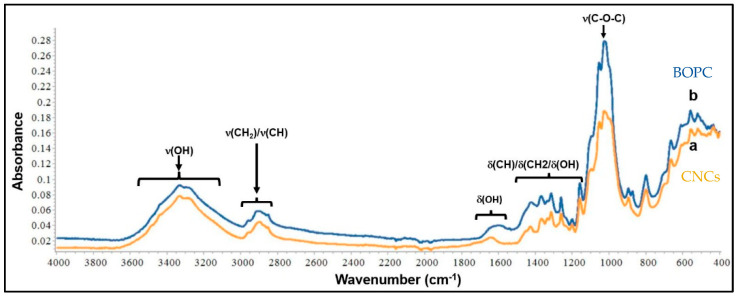
ATR/FT−IR spectra of (a) bleached orange peel cellulose (BOPC) and (b) cellulose nanocrystals (CNCs) with the assignment of the most prominent absorption bands.

ATR/FT-IR spectroscopy was used to analyze orange peel cellulose and CNCs to determine if sulfuric acid hydrolysis had significantly altered the chemical backbone of the polymer (Figure 6).

The stretching and bending vibrations of OH correspond to cellulose functionalities and minute amounts of water that have been absorbed (due to the hydrophilic nature of the fibers), which are represented by the bands between 3000 and 3600 cm^−1^ and the peak at approximately 1647 cm^−1^, respectively [19,49,50]. Peaks in the 1350–1500 cm^−1^ range mainly represent bands of CH/CH_2_ and OH bending vibrations, whereas peaks in the 2800–3000 cm^−1^ region can be attributed to stretching vibrations of CH/CH_2_ groups. [16,49]. Typical “finger-prints” of cellulose were also found at 1160 cm^−1^, 900 cm^−1,^ and 560 cm^−1^, showing the presence of β-glycosidic links in the structure of the cellulose and OH out-of-plane bending vibrations [51]. The peak at 1315 cm^−1^ arose from the bending vibrations of the CH and C-O groups of the rings in polysaccharides, while the very intense bands in the 1030–1160 cm^−1^ range corresponded to C-O-C stretching and C-H rocking of the pyranose ring [48]. No significant differences were identified between the two spectra, suggesting that the chemical structure was retained in the CNCs after acid hydrolysis. In both spectra, there were no peaks at 1735 cm^−1^, which are typically attributed to the C=O stretching of the uronic and acetyl ester groups of hemicelluloses as well as the ester linkages of carboxyl groups in p-coumaric and ferulic acids of hemicelluloses and lignin. Overall, these results indicated that the applied purification step, which included an alkali treatment, successfully removed noncellulosic contaminants such as hemicelluloses, pectin, and lignin from the raw matrix and helped in rearranging an important portion of its crystalline structure. The subsequent acid hydrolysis effectively reduced the amorphous character of cellulose without affecting its chemical structure and the crystalline domains. This effect was probably due to the hydronium ions, which could enter into the amorphous region of the fibers, resulting in the cleavage of cellulose glycoside bonds, thus liberating the crystals. These results were in accordance with those observed by Perumal et al. [52] for CNCs isolated from areca waste fibers.

**Figure 7:**
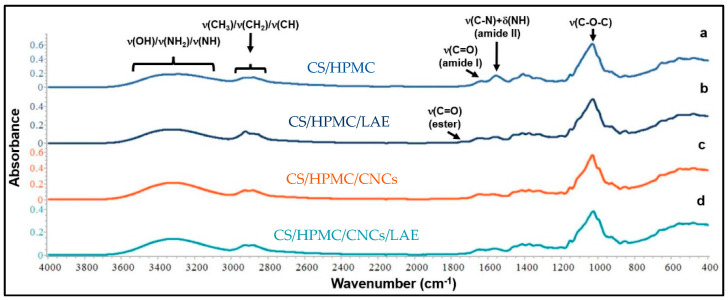
ATR/FT−IR spectra of films based on (a) chitosan/hydroxypropyl methylcellulose blend (CS/HPMC), (b) CS/HPMC/LAE^®^, (c) CS/HPMC/CNCs, and (d) CS/HPMC/CNCs/LAE^®^.

ATR/FT-IR spectroscopy was carried out to determine the spectroscopic changes brought by the addition of CNCs (10% *w*/*w* of biopolymer) and/or LAE^®^ (5% *w*/*w*) to the CS/HPMC film matrix (Figure 7). The pure CS/HPMC control film’s IR spectrum (Figure 7a) revealed the peaks that were connected to the amide-I and amide-II bands, respectively, at 1647 and 1557 cm^−1^. The stretching vibrations of the OH, NH, and NH_2_ groups were responsible for the broad absorption bands between 3600 and 3000 cm^−1^. The stretching vibrations of the HPMC CH_3_, CH_2_, and -CH groups were responsible for the band between 2800 and 3000 cm^−1^. The CH_3_/CH_2_ groups’ bending vibrations were found in the region between 1300 and 1500 cm^−1^. The saccharide structure can be attributed to the most intense bands between 930 and 1150 cm^−1^, which represent the ν(C-O-C) stretching vibrations of the ß-glycosidic links within the polymer structure. Overall, the spectroscopic data demonstrated the great compatibility of CS with HPMC to create biodegradable films [4,48].

Active films containing LAE^®^ (Figure 7b,d) revealed new absorption bands in the 2800–3000 cm^−1^ region and at 1739 cm^−1^, corresponding to the contributions of antisymmetric ν(CH_2_) and ν(C=O) stretching vibrations, respectively [53,54]. The shift of the amide II band from 1557 cm^−1^ to 1565 cm^−1^ (Figure 7b) and 1574 cm^−1^ (Figure 7d) confirmed that the molecular functionalities of LAE^®^, such as carbonyl, amino, and amino groups, worked with the hydroxyl, amino, and ether groups of the CS/HPMC mixture to produce antagonistic intermolecular interactions. Additionally, the intensified peak at 1645 cm^−1^ suggested that this additive contributed to creating amide C=O groups in the polymer matrix [30].

CNCs slightly affected the shape and intensity of the main bands of CS/HPMC blend films, while no new characteristic bands were observed (Figure 7c,d). It was found that the peak at 3346 cm^−1^ shifted to 3335 cm^−1^, indicating that there was hydrogen bonding between the CNCs’ hydroxyl groups and the OH, NH, NH_2_, and C=O groups of the CS/HPMC matrix [55]. The peak at 1557 cm^−1^ shifted to 1571 cm^−1^ and 1574 cm^−1^, confirming the interactions that occurred between the nanofillers and the polymer backbone [38]. These results were in accordance with those observed by Zeng et al. [56] for CS films enriched with CNCs isolated from pomegranate peels. Conversely, Costa et al. [48] observed the appearance of new bands (namely, the ones at 1369, 1315, and 1053 cm^−1^) related to the addition of CNCs to CS film-forming solutions. The different findings of the present studies may be related to the presence of HPMC in the film-forming solution, which is a derivative of cellulose, and thus could disguise the contribution of orange CNCs to the IR spectrum of the film in terms of new bands.

#### 3.3.3. Thickness and Mechanical Properties

For a material to be suitable for industrial packaging lines, it must meet the basic requirements of adequate mechanical strength and extensibility in order to keep the qualities of the food inside the packaging during processing, shipping, retailing, and storage. The thickness, elongation (E%), Young’s modulus (YM), and tensile strength (TS) of control and CNCs- and/or LAE^®^-enriched films are shown in Table 2. Typical stress-strain curves of the films tested in this study are reported in Figure S2 in Supplementary Materials.

The thickness ranged between 32.7 and 38.1 µm. The thickness of the control film was the lowest observed in this study. The reinforcement with CNCs (10% (*w*/*w*) of biopolymer) increased the thickness due to the higher solid content (*p* < 0.05) [48,57]. These results were in accordance with recent studies conducted on chitosan, PVA/chitosan [56], and gelatin films [58], which were enriched with different concentrations of cellulose-based nanofillers. The incorporation of LAE^®^ (5% (*w*/*w*) of biopolymer) slightly increased the thickness, probably due to its emulsifying behavior, which induced major retention of water molecules in the film matrix. Additionally, LAE^®^ might contribute to loosening the film matrix, reducing its homogeneity, and consequently increasing the thickness [59]. The co-presence of CNCs and LAE^®^ induced a further increase in thickness, which might be due to the synergistic effect of the two additives.

The maximum tension a film can withstand before breaking is measured by TS. According to conventional standards, the TS of films for packaging applications should be greater than 3.5 MPa [60]. In the present study, the control film based on a CS/HPMC blend had a TS of 17.5 MPa. The presence of CNCs considerably raised the TS of the films (*p* < 0.05) [16,61]. This behavior is explained by the abundance of hydroxyl functionalities on the CNC’s surface, which effectively interacts with the CS/HPMC mixture to generate hydrogen bonds [62] and fill the empty spaces between the polymer chains [63]. In this sense, the high aspect ratio of CNCs might positively affect the strength of the films by creating interfacial connections with the polymer matrix, generating a rigid and dense three-dimensional network (i.e., mimicking a crosslinking effect) [64,65]. Another contribution to this effect could be possibly given by the electrostatic interactions occurring among the cationic amine groups of chitosan and the anionic sulfate groups of CNCs [56]. LAE^®^ had a statistically irrelevant influence (*p* > 0.05) on the TS of CS/HPMC films. Conversely, Haghighi et al. [30] observed that LAE^®^ induced a slight decrease in the TS of CS/PVA blend films.

E% measures the stretchability of the films [66]. The CNCs’ inclusion did not affect the E% of the control film, which was 18.9% (*p* > 0.05). Gonzáles et al. [67] showed comparable results for soy protein films reinforced with cellulose nanofibers (0–10% (*w*/*w*) of biopolymer). The authors concluded that the addition of cellulose nanofibers primarily increased the TS and decreased the E% of the polymer matrix through a crosslinking process [15,63]. Additionally, Ali et al. [64] observed a negative effect of CNC addition on the extensibility of PVA/starch films. The authors concluded that the strong interactions between the matrix and filler particles could hinder the elongation of the films by retaining the shift between the polymer molecules. With respect to the control film, LAE^®^ improved its extensibility. This result can be attributed to LAE^®^’s emulsifying and plasticizing properties, which lowered the adhesion forces inside the polymer matrix [30,68].

YM quantifies the intrinsic stiffness of a film [61]. CS/HPMC control film showed a YM of 644.7 MPa, which increased to 705.8 MPa due to the presence of CNCs (*p* < 0.05) [57]. The high crystallinity of CNCs and the intra- and intermolecular interactions induced by the nanoparticle distribution inside the polymer matrix may be responsible for this effect, restricting biopolymer molecule mobility, and thus enhancing the stiffness of the films [48,56]. Overall, the results showed that CNC is a great reinforcement agent for CS/HPMC films. On the other hand, the inclusion of LAE^®^ significantly decreased (*p* < 0.05) the YM of CS/HPMC films (407.0 MPa). This result may be explained by the antagonistic interaction between the molecular functionalities of LAE^®^ and the CS/HPMC blend, which limited the cohesion forces between the polymers and lowered the level of physical crosslinking by reducing the intermolecular hydrogen bonds [30]. This effect was mitigated by the presence of both LAE^®^ and cellulose nanocrystals in CS/HPMC/CNCs/LAE^®^ films (529.1 MPa).

#### 3.3.4. Color

Table 3 lists the color characteristics (L*, a*, and b*) and total color difference (ΔE*) of films based on a CS/HPMC blend as the control, those reinforced with CNCs, and/or those enriched with LAE^®^.

The parameters L*, a*, and b* enable the evaluation of the film’s visual quality. L* (quantifying the film’s lightness) ranged from 97.5 to 95.6, indicating that all the films were bright. The incorporation of CNCs slightly decreased the L* (*p* < 0.05), while previous studies suggested that the addition of CNCs at different concentrations did not affect this parameter for starch-based films [15]. Meanwhile, the addition of LAE^®^ did not significantly affect this parameter (*p* > 0.05), as previously described by Haghighi et al. [30] in different studies [40]. For all the films presented in this study, the a* (which represents the greenness-redness color component) was negative. Both CNCs and LAE^®^ induced a slight increase in this value, corresponding to the film’s slight yellowish shade (*p* < 0.05). Upon the addition of CNCs and LAE^®^, the b* (expressing the blueness-yellowness color component) rose (*p* < 0.05) [15]. The film’s ΔE* ranged from 7.5 to 10.8, and both CNCs and LAE^®^ considerably changed this property (*p* < 0.05), as already demonstrated by Roy et al. [58] for gelatin-based films. Variations in b* and changes in film thickness, driven by the insertion of CNCs, could also contribute to this effect. Similar findings for polysaccharide-based films reinforced with plant-based CNCs were reported by Li et al. [16].

#### 3.3.5. UV Barrier, Light Transmittance, and Opacity Value

UV-Vis light barrier properties represent a key attribute of active films, protecting the packed products from photo-oxidation and avoiding the loss of nutritive components. Table 4 lists the UV-Vis light transmittance values in the 200–800 nm range as well as the opacity values of CS/HPMC blend films, those reinforced with CNCs, and those enriched with LAE^®^. With a light transmittance value of less than 0.1%, all films demonstrated outstanding barrier properties against UV-C radiation at 200 nm. While CNCs significantly enhanced barrier performance in the range of 280–350 nm UV-B and UV-A light due to their UV-shielding ability [56], LAE^®^ did not affect the percentage of light transmission in the aforementioned range. Similar results were reported by Sirviӧ et al. [35]. This effect may be explained by the reduction in light transmission caused by the dispersion of CNCs in the polymer matrix [16].

The visible range (400–800 nm) wavelengths were transmitted at a greater percentage than 80% for both the control and LAE^®^-containing films, demonstrating the exceptional transparency and brightness of CS/HPMC films. The transmission of visible light was not significantly impacted by the actual presence of LAE^®^. Conversely, the reinforcement of films by CNCs caused a slight decrease in film transparency. Similar results for hydrocolloid-based films reinforced with CNCs were reported by Costa et al. [48]. The authors concluded that the presence of CNCs induced a modest reduction in the transparency of the film because of their accumulation in the polymeric network, which led to light diffusion.

The opacity levels were in the range of 1.5–3.1. The control film exhibited the lowest value, confirming the high transparency of this film. The opacity was enhanced by the addition of CNCs (*p* < 0.05). This result was in accordance with those described by different authors [15,48,69]. These findings could be attributed to the nanofiller accumulation in the polymer matrix, causing light diffusion and scattering, and thus lower light transmittance [56]. A slightly different result was reported by Perumal et al. [52], who highlighted that the addition of CNCs to CS/PVA film-forming solutions did not cause a relevant increase in the film’s opacity. In this case, the authors justified the obtained results with the high degree of dispersion of CNCs in the film matrix, avoiding the formation of CNC aggregates and thus the lack of light scattering phenomena. The addition of LAE^®^ to CS/HPMC/CNCs film seemed to mitigate the effect of CNCs, reducing the opacity. This novel result suggests that this compound acted as an emulsifier, promoting a more homogeneous distribution of CNCs within the matrix and avoiding their agglomeration. Overall, all the films had opacity values strictly close to those measured for low-density polyethylene films and other commercial plastic materials intended for food applications [70]. Thus, the active films created in this work may offer a clear view of the food composition and condition, which is a significant factor in consumer acceptability [71].

#### 3.3.6. Water Content, Water Solubility, and Water Vapor Permeability

Sensitivity to water represents the main criticism related to biodegradable films. Biopolymers tend to absorb water when surrounded by a moist environment. The measurement of water-related properties represents a key step in tailoring a packaging system for targeted applications [72]. Table 5 shows the water content (WC), water solubility (WS), and water vapor permeability (WVP) of films based on a CS/HPMC blend as the control and those reinforced with CNCs and/or added with LAE^®^.

Values from 19.7 to 23.9% were obtained for WC. LAE^®^ addition induced the retention of water molecules in the film matrix, but not significantly from a statistical point of view (*p* > 0.05), as reported by Moreno et al. [54]. Conversely, the incorporation of CNCs slightly decreased this value, probably due to their strong interaction with the CS/HPMC network, which led to a reduction in the film hydrophilicity [67]. The WS of the control film was 52.5%. The addition of CNCs decreased this value to 35.6% (*p* < 0.05). This observation could be explained by the creation of hydrogen bonds between the CNCs and the hydroxyl groups of the CS/HPMC blend, restricting the movement of hydrophilic compounds toward water [73]. CS/HPMC films containing LAE^®^ showed the highest values of WS (62.5%). This effect is most likely due to LAE^®^’s restructuring behavior, which tends to bind water molecules, increasing the solubility of the film itself, as demonstrated by the lowest WVP value of this blend.

The ability of packaging to restrain the transmission of moisture from the environment to the packaged foodstuffs and vice versa is strongly important to maintaining the quality of food [74]. Both fresh and dry products take advantage of this feature since the former avoids dehydration and the latter avoids moisture uptake [75]. The hydrophilic character of the film, the co-presence of crystalline and amorphous zones, and the mobility of the polymer chains are only a few of the variables that affect the WVP of packaging. The control film had a WVP of 7.2 (g·mm/day·kPa·m^2^). The addition of CNCs reduced the WVP value to 5.8 (g·mm/day·kPa·m^2^), which is a reduction of 19.5%. This result confirmed that the addition of CNCs represents an effective strategy to counteract the film’s sensitivity to moisture. In fact, their homogeneous dispersion into the FFS decreased the availability of free hydroxyl groups inside the polymer matrix and generated a three-dimensional frame that slowed down the diffusion of water molecules [3]. The WVP of CS/HPMC films, on the other hand, was dramatically raised by LAE^®^ to 9.5 (g·mm/day·kPa·m^2^). The WVP value of CS/HPMC/CNCs/LAE^®^ film was 6.8 (g·mm/day·kPa·m^2^). These findings could be explained by the partial hydrogen bond breakdown caused by LAE^®^, which encouraged the creation of amorphous patterns inside the polymer [76].

#### 3.3.7. In Vitro Antimicrobial Activity

When food is produced, processed, or stored, it is extremely vulnerable to microbial contamination and deterioration. Food spoilage is largely caused by bacteria, which can also have detrimental impacts on human health. Food products are highly susceptible to microbial spoilage, which can occur during production, processing, and storage. Bacteria are mainly responsible for food deterioration and can induce harmful effects on human health [48]. Thus, biobased active films with antimicrobial properties represent effective tools for avoiding the proliferation of spoilage and pathogenic bacteria.

The disk diffusion assay was used to examine the antibacterial activity of CS/HPMC blend films against *E. coli*, *P. fluorescens*, *L. monocytogenes*, and *S. enterica* sv. Typhimurium, as well as those reinforced with CNCs and/or enriched with LAE^®^ (Table 6). The studied micro-organism’s development was unaffected by the LAE^®^-free films. All the examined bacterial growth was suppressed by active films supplemented with LAE^®^ bacteria [77]. The cationic surfactant action of LAE^®^ on the cytoplasmic membrane of gram-positive and gram-negative bacteria has been widely linked to its antibacterial activity. This compound affects the membrane’s potential and the cytoplasm’s permeability, which inhibits cell development and causes viability loss [54]. Furthermore, LAE^®^ has been reported to induce changes in the DNA structure, causing aggregations through ionic bridges [78]. Compared to *E. coli* and *S. Typhimurium*, LAE^®^ showed slightly higher efficacy against *L. monocytogenes*. The authors concluded that gram-negative bacteria are generally less vulnerable to the effects of antimicrobial substances because they have an outer membrane that covers their cell wall and prevents hydrophobic substances from diffusing through [1,79,80]. The high density of the *S.* Typhimurium cell membrane, which is related to the high content of phospholipid components, such as phosphatidylethanolamine and phosphatidylglycerol, may be the cause of the pathogen’s good resistance to antimicrobial molecules that act on the membrane [81]. Despite being gram-negative, *P. fluorescens* proved to be the most susceptible among the studied micro-organisms. This might be explained by the absence of defenses against oxidative stress, which results in a general decline in cell viability. Due to the dense nanoscale structure created by the presence of CNCs, which could retain LAE^®^ and prevent its migration in the external medium, CS/HPMC/LAE^®^ film had better antibacterial efficacy than CS/HPMC/CNCs/LAE^®^ film.

## 4. Conclusions

The purpose of this work was to provide an extraction method for obtaining CNCs from leftover orange peels using an alkaline/H_2_O_2_ bleaching process and sulfuric acid hydrolysis. The extracted CNCs were used as a reinforcing agent to produce nanocomposite films for food packaging applications based on the CS/HPMC blend, which were enriched with LAE^®^ to confer antimicrobial activity.

Except for the slight agglomeration of CNCs, SEM revealed that both CNCs and LAE^®^ were equally dispersed in the polymer matrix to create homogeneous films, demonstrating high compatibility between the polymers and the additives. The establishment of hydrogen bonds between the biopolymer network’s hydroxyl and amino groups and the functional groups of CNCs and LAE^®^ was confirmed by the ATR/FT-IR spectra. While all of the film’s opacity values remained below five, CNCs considerably enhanced the UV and light barrier qualities of the films, which may be effective in protecting food from photo-oxidation and UV deterioration. The addition of CNCs significantly improved the tensile strength by 45% compared to the control film and increased stiffness, while WS and WVP were reduced due to the hydrogen bonding linkages formed within the polymer matrix. In contrast, LAE^®^ addition improved the elasticity by 26% due to its emulsifying behavior and inhibited the growth of all the tested food-borne bacterial pathogens, including *S. enterica* subsp. Typhimurium, *E. coli*, *L. monocytogenes*, and *P. fluorescens*. Films containing both LAE^®^ and CNCs showed improvements for all the tested parameters. Overall, these findings suggest the possibility of employing these films as a green substitute to conventional plastic materials for packaging foods sensitive to microbiological decay and photo-oxidation. Furthermore, the extraction of CNCs from agrifood waste and their subsequent application as nanoreinforcing agents may represent a feasible strategy for minimizing the environmental and economic costs related to waste disposal, thus creating a new opportunity for businesses with high growth potential.

## Figures and Tables

**Table 1:** Crystallinity index of the films and CNCs of orange peel was calculated as described in Section 2.6.

Film Sample	Crystallinity Index %
CS/HPMC	7.65 ± 0.06 ^a^
CS/HPMC/LAE^®^	7.42 ± 0.13 ^a^
CS/HPMC/CNCs	24.96 ± 1.05 ^b^
CS/HPMC/CNCs/LAE^®^	26.81 ± 1.36 ^b^
CNC orange peel	61.93 ± 3.46 ^c^

Data are presented as mean ± standard deviation (*n* = 10). Significant differences (*p* < 0.05) are indicated by different letters in the same column.

**Table 2:** Thickness, tensile strength (TS), elongation (E%), and Young’s modulus (YM) of films based on chitosan/hydroxypropyl methylcellulose blend (CS/HPMC) as control and those reinforced with cellulose nanocrystals (CNCs 10% (*w*/*w*) of biopolymer) and/or enriched with lauroyl arginate ethyl (LAE^®^ 5% (*w*/*w*) of biopolymer).

Film Sample	Thickness (µm)	TS (MPa)	E (%)	YM (MPa)
CS/HPMC	32.7 ± 2.0 ^a^	17.5 ± 1.0 ^a^	18.9 ± 0.9 ^a^	644.7 ± 25.2 ^c^
CS/HPMC/CNCs	35.3 ± 1.8 ^b^	25.4 ± 2.7 ^b^	19.9 ± 1.6 ^a^	705.8 ± 57.2 ^d^
CS/HPMC/LAE^®^	34.1 ± 2.6 ^ab^	15.1 ± 0.9 ^a^	23.9 ± 1.0 ^b^	407.0 ± 28.9 ^a^
CS/HPMC/CNCs/LAE^®^	38.1 ± 1.8 ^c^	26.4 ± 1.4 ^b^	27.8 ± 1.9 ^c^	529.1 ± 48.8 ^b^

Data are presented as mean ± standard deviation (*n* = 10). Significant differences (*p* < 0.05) are indicated by different letters in the same column.

**Table 3:** Color parameters (L*, a*, and b*) and total color variation (ΔE*) of films based on chitosan/hydroxypropyl methylcellulose blend (CS/HPMC) as control and those reinforced with cellulose nanocrystals (CNCs 10% (*w*/*w*) of biopolymer) and/or enriched with lauroyl arginate ethyl (LAE^®^ 5% *w*/*w* of polymer).

Film Sample	Color Parameters			
	L*	a*	b*	ΔE
CS/HPMC	97.2 ± 0.3 ^c^	−1.1 ± 0.1 ^a^	7.2 ± 0.6 ^a^	7.5 ± 0.6 ^a^
CS/HPMC/CNCs	95.6 ± 0.2 ^a^	−0.9 ± 0.06 ^b^	10.2 ± 0.4 ^c^	10.8 ± 0.4 ^b^
CS/HPMC/LAE^®^	97.5 ± 0.3 ^c^	−0.8 ± 0.06 ^c^	7.5 ± 0.6 ^a^	7.8 ± 0.7 ^a^
CS/HPMC/CNCs/LAE^®^	96.3 ± 0.2 ^b^	−0.9 ± 0.03 ^b^	8.9 ± 0.8 ^b^	10.0 ± 1.0 ^b^

Data are presented as mean ± standard deviation (*n* = 10). Significant differences (*p* < 0.05) are indicated by different letters in the same column.

**Table 4:** UV-Vis light transmittance (%) and opacity of the films based on chitosan/hydroxypropyl methylcellulose blend (CS/HPMC, 2% *w*/*v*) as the control and reinforced with cellulose nanocrystals (CNCs, 10% *w*/*w* of biopolymer) and/or enriched with lauroyl arginate ethyl (LAE^®^, 5% *w*/*w* of biopolymer).

Film Sample	Light Transmission (%) at Different Wavelengths (nm)								Opacity (600 nm)
	200	280	350	400	500	600	700	800	
CS/HPMC	<0.1	25.5	45.7	69.2	83.2	87.2	89.4	90.2	1.5 ± 0.1 ^a^
CS/HPMC/CNCs	<0.1	12.4	31.8	54.4	67.2	73.1	76.2	78.0	3.1 ± 0.2 ^d^
CS/HPMC/LAE^®^	<0.1	26.8	51.2	71.7	83.7	87.4	88.7	89.4	1.9 ± 0.06 ^b^
CS/HPMC/CNCs/LAE^®^	<0.1	22.3	44.6	64.4	76.8	81.2	83.8	85.1	2.4 ± 0.2 ^c^

Data are presented as mean ± standard deviation (*n* = 10). Significant differences (*p* < 0.05) are indicated by different letters in the same column.

**Table 5:** Water content (WC), water solubility (WS), and water vapor permeability (WVP) of films based on chitosan/hydroxypropyl methylcellulose blend (CS/HPMC, 2% *w*/*v*) as the control and those reinforced with cellulose nanocrystals (10% *w*/*w* of biopolymer) and/or lauroyl arginate ethyl (LAE^®^, 5% *w*/*w* of biopolymer).

Film Sample	WC (%)	WS (%)	WVP (g·mm/kPa·Day·m^2^)
CS/HPMC	22.6 ± 2.6 ^b^	52.5 ± 1.8 ^b^	7.2 ± 0.5 ^b^
CS/HPMC/CNCs	19.7 ± 0.8 ^a^	35.6 ± 0.8 ^a^	5.8 ± 0.3 ^a^
CS/HPMC/LAE^®^	23.9 ± 1.1 ^b^	62.5 ± 1.2 ^d^	9.5 ± 0.7 ^c^
CS/HPMC/CNCs/LAE^®^	22.8 ± 0.7 ^b^	56.9 ± 1.0 ^c^	6.8 ± 0.5 ^ab^

Data are presented as mean ± standard deviation (*n* = 10). Significant differences (*p* < 0.05) are indicated by different letters in the same column.

**Table 6:** Inhibition zone diameters (expressed in mm) of the film disks (18 mm diameter) based on chitosan-hydroxypropyl methylcellulose blend (CS/HPMC) as the control and those reinforced with cellulose nanocrystals (CNCs 10% *w*/*w* of biopolymer), and/or enriched with lauroyl arginate ethyl (LAE^®^ 5% *w*/*w* of biopolymer).

Film Sample	*S. enterica*	*E. coli*	*L. monocytogenes*	*P. fluorescens*
CS/HPMC	N.D.	N.D.	N.D.	N.D.
CS/HPMC/CNCs	N.D.	N.D.	N.D.	N.D.
CS/HPMC/LAE^®^	0.9 ± 0.09 ^bA^	4.4 ± 0.4 ^bB^	6.7 ± 0.6 ^aC^	8.5 ± 0.7 ^bD^
CS/HPMC/CNCs/LAE^®^	0.5 ± 0.07 ^aA^	3.4 ± 0.4 ^aB^	6.5 ± 0.6 ^aD^	5.7 ± 0.4 ^aC^

Values are presented as mean ± standard deviation (*n* = 3). N.D. means not detected. Different lowercase letters in the same column indicate significant differences (*p* < 0.05). Different capital letters in the same row indicate significant differences (*p* < 0.05).

## Data Availability

All related data and methods are presented in this paper. Additional inquiries should be addressed to the corresponding author.

## Supplementary Materials

The following supporting information can be downloaded at: https://www.mdpi.com/article/10.3390/foods12050960/s1, Figure S1: Production process of CNCs from wasted orange peels; Figure S2: Stress-strain curves.
