# Correction: Bigi et al. Waste Orange Peels as a Source of Cellulose Nanocrystals and Their Use for the Development of Nanocomposite Films. *Foods* 2023, *12*, 960

**DOI:** 10.3390/foods12091902

**Published:** 2023-05-06

**Authors:** Francesco Bigi, Enrico Maurizzi, Hossein Haghighi, Heinz Wilhelm Siesler, Fabio Licciardello, Andrea Pulvirenti

**Affiliations:** 1 Department of Life Sciences, University of Modena and Reggio Emilia, 42015 Reggio Emilia, Italy; francesco.bigi@unimore.it (F.B.); fabio.licciardello@unimore.it (F.L.); 2 Department of Physical Chemistry, University of Duisburg-Essen, 45141 Essen, Germany; 3 Interdepartmental Research Centre for the Improvement of Agri-Food Biological Resources (BIOGEST-SITEIA), University of Modena and Reggio Emilia, 42015 Reggio Emilia, Italy

## Text Correction

In the original publication [1], the authors wish to change “LAE” into “LAE^®^” in the full text. The changes are as follows:

In the abstract, change “LAE” into “LAE^®^”. There are four occurrences.

In the last paragraph of the introduction, change “LAE” into “LAE^®^”. There are two occurrences.

In Section 2.1, change “LAE” into “LAE^®^”. There is one occurrence.

In Section 2.5, change “LAE” into “LAE^®^”. There are three occurrences.

In Section 3.2 (including Table 1), change “LAE” into “LAE^®^”. There are three occurrences.

In the Section 3.3.1 (including Figures 4 and 5), change “LAE” into “LAE^®^”. There are nine occurrences.

In Section 3.3.2 (including Figure 6), change “LAE” into “LAE^®^”. There are five occurrences.

In Section 3.3.3 (including Table 2), change “LAE” into “LAE^®^”. There are fifteen occurrences.

In Section 3.3.4 (including Table 3), change “LAE” into “LAE^®^”. There are eight occurrences.

In Section 3.3.5 (including Table 4), change “LAE” into “LAE^®^”. There are eight occurrences.

In Section 3.3.6 (including Table 5), change “LAE” into “LAE^®^”. There are ten occurrences.

In Section 3.3.7 (including Table 6), change “LAE” into “LAE^®^”. There are twelve occurrences.

In the conclusion, change “LAE” into “LAE^®^”. There are five occurrences.

The authors apologize for any inconvenience caused and state that the scientific conclusions are unaffected. This correction was approved by the Academic Editor. The original publication has also been updated.
