# Supplementary figures and images for: Waste Orange Peels as a Source of Cellulose Nanocrystals and Their Use for the Development of Nanocomposite Films
Source: Foods. 2023 Feb 24;12(5):960. doi: 10.3390/foods12050960 (PMC10001245; doi:10.3390/foods12050960)

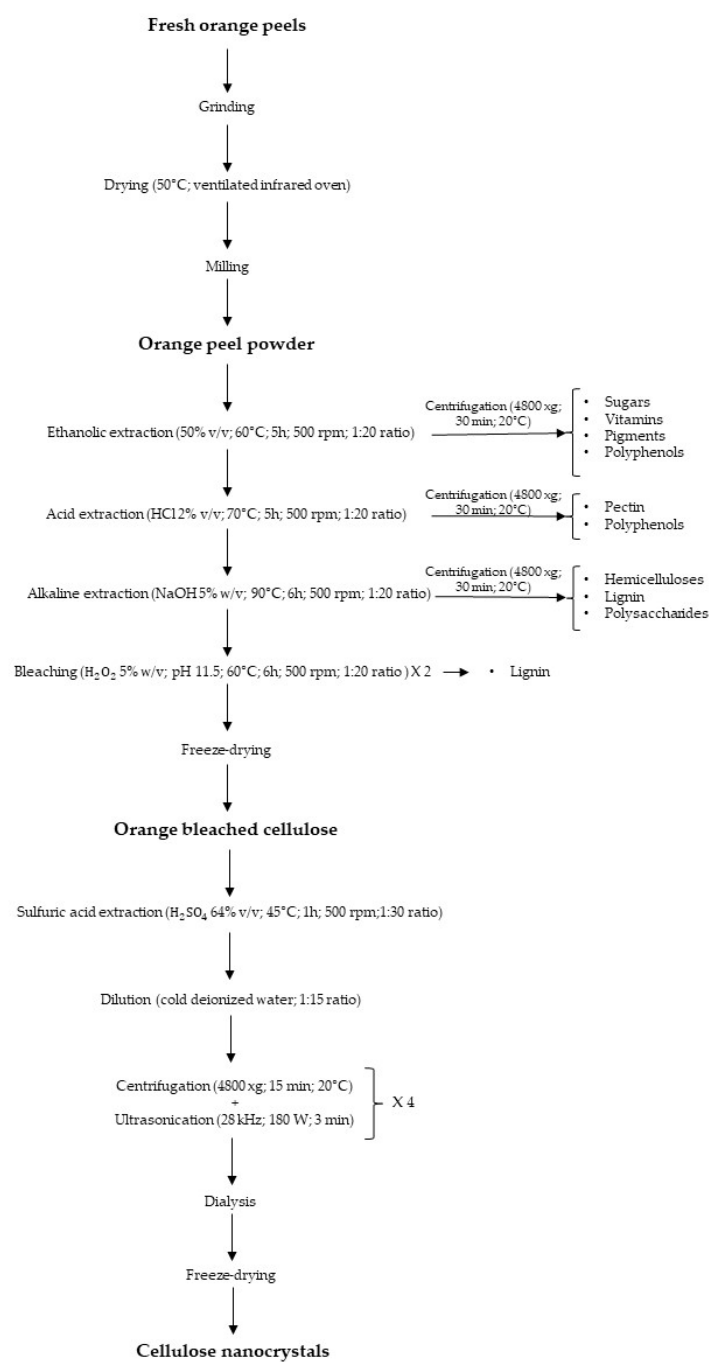

**Figure S1: Production process of CNCs from wasted orange peels**

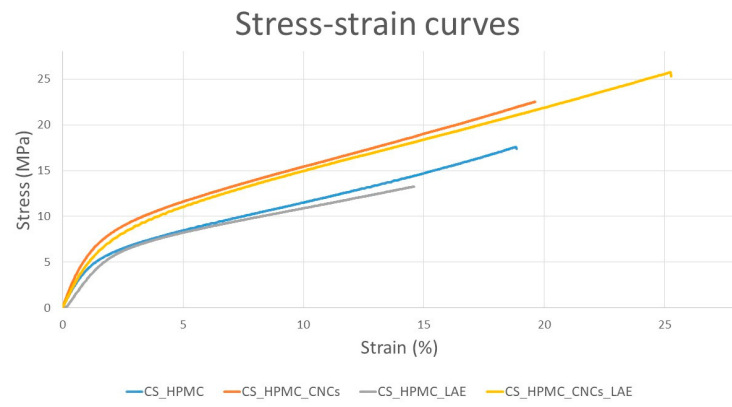

**Figure S2: Stress-strain curves.**

Supplement: Supplementary file 1 [file foods-12-00960-s001.zip › foods-2200079-supplementary.pdf]
